# RISK-GPT: Using ChatGPT to construct a reliable risk factor database for all known diseases

**DOI:** 10.7189/jogh.13.03037

**Published:** 2023-08-04

**Authors:** Xi Chen, Xin Zhang, Yuan Liu, Ziyuan Wang, Yixin Zhou, Ming Chu

**Affiliations:** 1 Department of Adult Joint Reconstructive Surgery, Beijing Jishuitan Hospital, Capital Medical University, Beijing, China; 2 Department of Immunology, School of Basic Medical Sciences, Peking University. NHC Key Laboratory of Medical Immunology, Peking University, Beijing, China; 3 Institute of Computing and Intelligence, Harbin Institute of Technology (Shenzhen), HIT Campus of University Town of Shenzhen, Shenzhen, China; 4 Central Laboratory, Peking University School of Stomatology, Beijing, China

**Figure:**
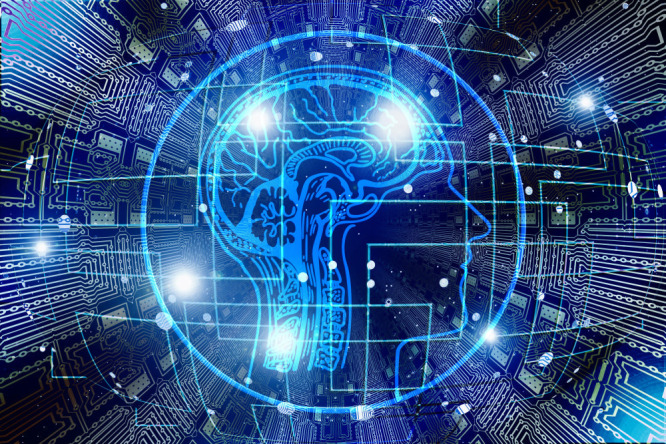
Photo: Artificial intelligence, brain, thinking picture. Source: Pixabay, free to use (https://pixabay.com/zh/illustrations/artificial-intelligence-brain-think-3382507/).

It is widely acknowledged that a comprehensive understanding of the risk factors associated with the disease is vital for both the general public and medical researchers [1,2]. For the population at large, understanding disease risk factors can enable informed decisions about lifestyle choices and preventative measures, thus contributing to overall health and well-being [3,4]. With knowledge of the disease risk factors, individuals can consciously strive to mitigate their risk, adopt healthier behaviours, and reduce the incidence of preventable diseases. For medical researchers, studying disease risk factors forms the basis of epidemiological investigations [5] that elucidates the complex aetiology of diseases, aiding in identifying causative and contributing factors; consequently, this guides the development of targeted therapeutic approaches, provides clinical guidelines, and drives innovative research on disease prevention and treatment [6]. Moreover, a comprehensive understanding of disease risk factors enables the conception of population-specific intervention strategies that contribute to health equity and the broader goal of global health improvement [7].

However, as one of the most popular topics, the literature on disease-related risk factors has increased sharply over the last 20 years from 332 182 (2002) to 1 748 504 (2022). Given that over 120 000 studies were published in a single year, it is impossible to finish reading them all, which exerts an information overload on researchers. These requirements encouraged us to apply a natural language processing (NLP) algorithm to extract risk factor information from numerous studies.

The advent of large language models (LLM) such as ChatGPT heralds a new era of automation and cognitive assistance, reshaping engagement with information and decision-making processes [8]. The ChatGPT model has gained recognition for its ability to extract information, demonstrate an in-depth understanding of context, and provide valuable insights across various subjects [9]. This study aimed to develop and evaluate four risk factor extraction models based on ChatGPT.

In this study a risk factor-related literature database including 1 748 504 papers was developed based on the literature downloaded from PubMed FTP (https://ftp.ncbi.nlm.nih.gov/pubmed/). For each test in the four models, we randomly selected 100 literature abstracts from the database and input them into ChatGPT to extract risk factor information according to the stipulations of various models.

Model 1 employed ChatGPT to extract risk factor information from the entire abstract, whereas Model 2 harnessed ChatGPT to extract risk factor details from the results section of the abstract. Model 3 filtered the input literature and extracted risk factors exclusively from the abstracts of clinical studies. Model 4 restricted itself to extracting risk factor information exclusively from the results section within clinical research abstracts.

**Figure 1:**
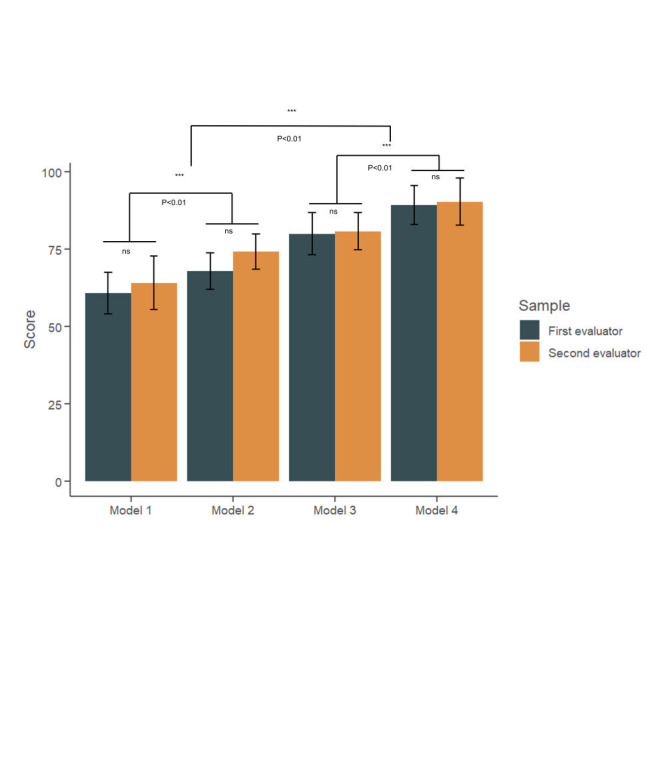
The accuracy score of four different models. The green bar represents the accuracy score from the first evaluator, while the orange bar is the score of the second evaluator. ns, represents no significant difference. ***, represents significant change (*P* < 0.01). In Model 1, 100 abstracts were randomly selected. In Model 2, the results section of 100 abstracts were analysed. In Model 3, 100 abstracts from clinical studies were selected. In Model 4, the results section of 100 clinical studies abstracts were analysed. These models were tested independently three times each.

Each model was tested independently in three iterations, and the results were verified by two experts. The performance of the models was gauged on a scale of 0 to 1, with 1 representing complete accuracy and 0 representing total inaccuracy (**Figure 1**). There were no significant differences between the different evaluation criteria in these four models (*P* > 0.05), implying that the assessment outcomes were consistent and accurate. However, significant differences were observed in the accuracy scores between the models. Model 1, which used the entire abstract for risk factor information extraction, only yielded an average accuracy of 54.4 ± 4.6. The average accuracy for model 2 increased to 65.3 ± 8.9 when the results section of abstracts was analysed. Upon further criteria restriction to clinical research abstracts, the average accuracy values for models 3 and 4 significantly increased, with accuracy values of 82.1 ± 7.8 and 92.6 ± 2.1, respectively.

We used stringent criteria for each literature review. Our data showed that ChatGPT has the potential to extract a plethora of data on disease risk factors from article abstracts.

In this study, Model 1 achieved an average accuracy score of only 54 ± 4.6. Upon further examination by the researchers assigned to review each article, the low score was attributed to contextual inferences in data that contained no evidence-based support. Therefore, we restricted the input information to the results section of the abstract of each article, which yielded significantly better accuracy in Model 2 (*P* < 0.01). Additionally, most of the inaccuracies observed in Model 2 were attributed to basic experimental articles; because many basic studies do not contain risk factor information, ChatGPT either extrapolates or fabricates risk factors from some of the abstracts. Further restrictions placed in Models 3 and 4 resulted in a significant increase in accuracy (*P* < 0.01), with Model 4 showing an accuracy of approximately 93.

Therefore, our study shows that ChatGPT can potentially be used to develop a RISK-GPT database for diseases. However, human validation is necessary to ensure accuracy [10]. Furthermore, even though artificial intelligence can assist humans in these matters, humans are responsible for its development and implementation [11].

This study had some limitations. First, the number of literature samples tested was small and should be drastically increased in future studies. Second, these ChatGPT models were not specifically designed to extract information from the medical literature. Furthermore, the results of the included studies were inconsistent within each model. There remains ample room for improvement to resolve these issues and better optimize ChatGPT to produce and develop a RISK-GPT database.

In conclusion, our study demonstrates that ChatGPT has the potential to extract disease risk factor data from article abstracts. However, limitations such as low accuracy in Model 1 and inconsistencies in the results indicate the need for further optimization and human validation to develop a reliable RISK-GPT database for diseases, emphasizing the responsibility of humans in its development and implementation.
